# Regenerative Endodontic Procedures in Immature Permanent Teeth: Biological Mechanisms and Clinical Outcomes

**DOI:** 10.7759/cureus.109575

**Published:** 2026-05-24

**Authors:** Abdullah Alqedairi

**Affiliations:** 1 Division of Endodontics, Department of Restorative Dental Sciences, College of Dentistry, King Saud University, Riyadh, SAU

**Keywords:** apexification, immature permanent teeth, narrative review, platelet-rich plasma, pulp regeneration, regenerative endodontics, revascularization, stem cells of the apical papilla

## Abstract

Necrotic immature permanent teeth with open apices pose a difficult management problem in clinical endodontics. Conventional apexification techniques resolve infection but do not support continued root development, leaving the tooth structurally vulnerable. Regenerative endodontic procedures (REPs) have emerged as a biologically oriented alternative that harnesses stem cells, scaffolds, and growth factors to form new tissue within the root canal space and encourage root maturation. This narrative review discusses the biological principles underlying REPs, the clinical protocols currently in use, the reported treatment outcomes, the key prognostic factors, and the limitations of current evidence. Overall, REPs demonstrate favorable rates of periapical healing and continued root development, with platelet-rich plasma and platelet-rich fibrin scaffolds showing advantages over blood clot alone for dentinal wall thickening. Pulp sensibility recovery is variable and does not reliably confirm true pulp regeneration. Current outcomes more closely reflect biologically supported repair than consistent, predictable pulp regeneration. Standardized protocols and longer-term follow-up studies are needed to fully establish the evidence base for these procedures.

## Introduction and background

Immature permanent teeth with pulp necrosis and apical periodontitis are among the more demanding cases encountered in endodontic practice. An incompletely formed root has thin dentinal walls, a wide open apex, and a short root length, any of which can compromise structural integrity and make conventional endodontic treatment difficult or impossible. Without treatment, these teeth face a high probability of cervical fracture, failed apical seal, and eventual extraction. The challenge is compounded by the fact that these cases predominantly occur in children and adolescents, where tooth loss carries significant functional, esthetic, and psychological consequences over a lifetime.

For many years, apexification using calcium hydroxide or mineral trioxide aggregate (MTA) was the accepted approach. Both methods can clear infection and create an apical stop, but neither supports further root development. The tooth remains non-vital with a structurally weak root. Long-term calcium hydroxide treatment also carries a recognized risk of increasing root fragility through its sustained high-pH effect on dentinal collagen [1]. MTA apical plug techniques, while faster and more predictable, similarly leave the tooth unable to complete its development, maintaining thin dentinal walls susceptible to fracture and limiting the long-term prognosis of the tooth.

Regenerative endodontic procedures developed from research into dental stem cell biology and tissue engineering. The procedure applies the well-established principle that tissue formation depends on three components: a viable cell source, a suitable scaffold, and appropriate biological signals. The early case reports by Banchs and Trope in 2004 showed that thorough disinfection followed by induction of a blood clot into the canal could stimulate striking root development in young patients, and this sparked sustained interest in the approach [2]. Both the American Association of Endodontists (AAE) and the European Society of Endodontology (ESE) now recommend REPs as the treatment of choice for necrotic immature permanent teeth when patient factors allow [3].

The clinical evidence accumulated over two decades has been encouraging. Periapical healing rates of 85-94% have been consistently reported across diverse study populations and protocols. Continued root development, encompassing apical closure, root lengthening, and dentinal wall thickening, is achieved in 74-81% of cases. Platelet-rich plasma and platelet-rich fibrin scaffolds have demonstrated superior dentinal wall thickening compared to blood clot alone in controlled trials. Pulp sensibility recovery is the least predictable outcome, reported in 25-70% of cases, and must be interpreted with caution as it does not reliably confirm true pulp tissue formation [4].

Questions remain, however, about the exact nature of the tissue that forms inside treated canals, how consistently favorable outcomes can be achieved, and what the long-term prognosis of treated teeth looks like. The clinical results we observe may sometimes reflect a form of biological repair rather than true regeneration of the pulp-dentin complex [4]. This review discusses the biological basis of REPs, the protocols currently in use, the available outcome data, the limitations of the evidence, and areas where further research is needed.

This narrative review was conducted through a literature review of PubMed/MEDLINE, Scopus, and Web of Science, covering publications from 2004 to 2025. Search terms included: regenerative endodontics, revascularization, immature permanent teeth, pulp regeneration, stem cells of the apical papilla, platelet-rich plasma, platelet-rich fibrin, and apexification. Studies were selected based on clinical relevance, methodological quality, and contribution to the understanding of REP outcomes. Inclusion was not restricted by study design; original research articles, randomized controlled trials, prospective and retrospective cohort studies, systematic reviews, meta-analyses, and key position statements from professional organizations were all considered. As this is a narrative rather than a systematic review, a formal Preferred Reporting Items for Systematic Reviews and Meta-Analyses (PRISMA) flowchart and risk-of-bias assessment were not conducted; instead, representative studies were selected to illustrate the breadth and development of the evidence base from early case reports through to recent large-scale investigations.

## Review

Biological basis of regenerative endodontic procedures

Stem Cell Sources and the Apical Papilla

The apical papilla is a loose connective tissue at the apex of developing roots. It carries a high concentration of stem cells, referred to as stem cells of the apical papilla (SCAP), and tends to survive pulp necrosis because its location apical to the main foramen keeps it outside the main zone of bacterial contamination. SCAPs have a higher proliferative rate than dental pulp stem cells, express standard mesenchymal markers (STRO-1, CD73, CD90, CD105), and can give rise to odontoblast-like cells when the right signals are present [5,6]. This makes them the main cell source responsible for root development after REP.

Other cell populations also contribute. Dental pulp stem cells (DPSCs) may survive in the apical portion of the canal in cases of partial necrosis. Periodontal ligament stem cells (PDLSCs) can migrate from surrounding periapical tissues, especially in teeth with periapical bone loss. Bone marrow-derived mesenchymal stem cells may also be drawn into the canal when bleeding is induced [7]. The exact share of each population in the final regenerated tissue has not been established, and this uncertainty helps explain why the histological findings from treated teeth vary so much between cases.

Scaffolds and Growth Factors

Whatever scaffold is placed in the canal must support cell attachment, proliferation, and differentiation while degrading at a rate compatible with tissue formation. The blood clot, produced by over-instrumentation of periapical tissues to cause controlled bleeding, remains the most widely used option. It forms a fibrin network containing transforming growth factor beta (TGF-beta), platelet-derived growth factor (PDGF), vascular endothelial growth factor (VEGF), and bone morphogenetic proteins (BMPs) released from activated platelets [8]. The main drawback is that its composition differs between patients, which is one likely reason for the inconsistency seen in outcomes.

Platelet-rich plasma (PRP) and platelet-rich fibrin (PRF) are prepared from the patient's own blood by centrifugation and contain concentrated growth factors delivered in a more controlled and reproducible way [9,10]. Several clinical trials have found that teeth treated with PRP or PRF show greater dentinal wall thickening than those treated with a blood clot scaffold [11]. PRF releases its growth factors more slowly due to the structure of its fibrin matrix, which may prolong the stimulatory effect on stem cells. Injectable PRF (i-PRF), a liquid form of PRF, has attracted interest in regenerative dentistry for its improved handling properties and cell-supportive capacity [12]. The final ethylenediaminetetraacetic acid (EDTA) rinse at the end of the disinfection sequence also contributes by demineralizing the canal wall surface and releasing growth factors, including TGF-beta1, fibroblast growth factor 2 (FGF-2), and BMP-2, that had been bound within the dentinal matrix [13,14].

Clinical protocols

Patient Selection and First Visit

Regenerative endodontic procedures are best suited to patients under 18 years of age with necrotic immature permanent teeth [3,4,7]. Teeth with canal walls too thin for safe rotary instrumentation, or with apices too wide to obturate conventionally, are especially good candidates. The procedure is not appropriate in cases of allergy to antibiotic paste components, teeth with irreparable structural damage, or patients unable to cooperate with a multi-visit course of treatment.

At the first visit, the priority is thorough disinfection while preserving as much dentinal structure as possible. Sodium hypochlorite is used at 1.5-3% rather than the 5.25% concentration used in conventional root canal treatment, as higher concentrations have been shown to reduce SCAP viability [13]. After irrigation, a final rinse with 17% EDTA prepares the canal wall surface and releases growth factors from within the dentine [14]. An intracanal medicament is then placed. The double antibiotic paste (DAP), containing ciprofloxacin and metronidazole, is generally preferred over the original triple antibiotic paste (TAP) because it avoids the grey-brown crown discoloration caused by minocycline [7]. Calcium hydroxide is a suitable alternative for practitioners wishing to avoid antibiotics altogether.

Second Visit and Coronal Seal

The second appointment is scheduled 2-4 weeks later, once the patient is symptom-free. The canal is re-irrigated with 17% EDTA, and the medicament is removed. A sterile file is then passed 2 mm beyond the apex to induce periapical bleeding, and the blood clot is allowed to form up to approximately 3 mm below the cementoenamel junction. Some clinicians place PRP or PRF instead of, or alongside, the blood clot [10]. A 3-4 mm layer of MTA or Biodentine® (Septodont, Saint-Maur-des-Fossés, France) is placed over the scaffold as a cervical barrier, and the tooth is then restored with a bonded composite. Biodentine has become widely used because it sets faster than MTA, handles more easily, and does not cause crown discoloration, which is an important consideration in anterior teeth [15]. The final restoration quality is considered one of the strongest predictors of long-term success, since any gap in the coronal seal risks recontamination of the canal [16].

**Figure 1:**
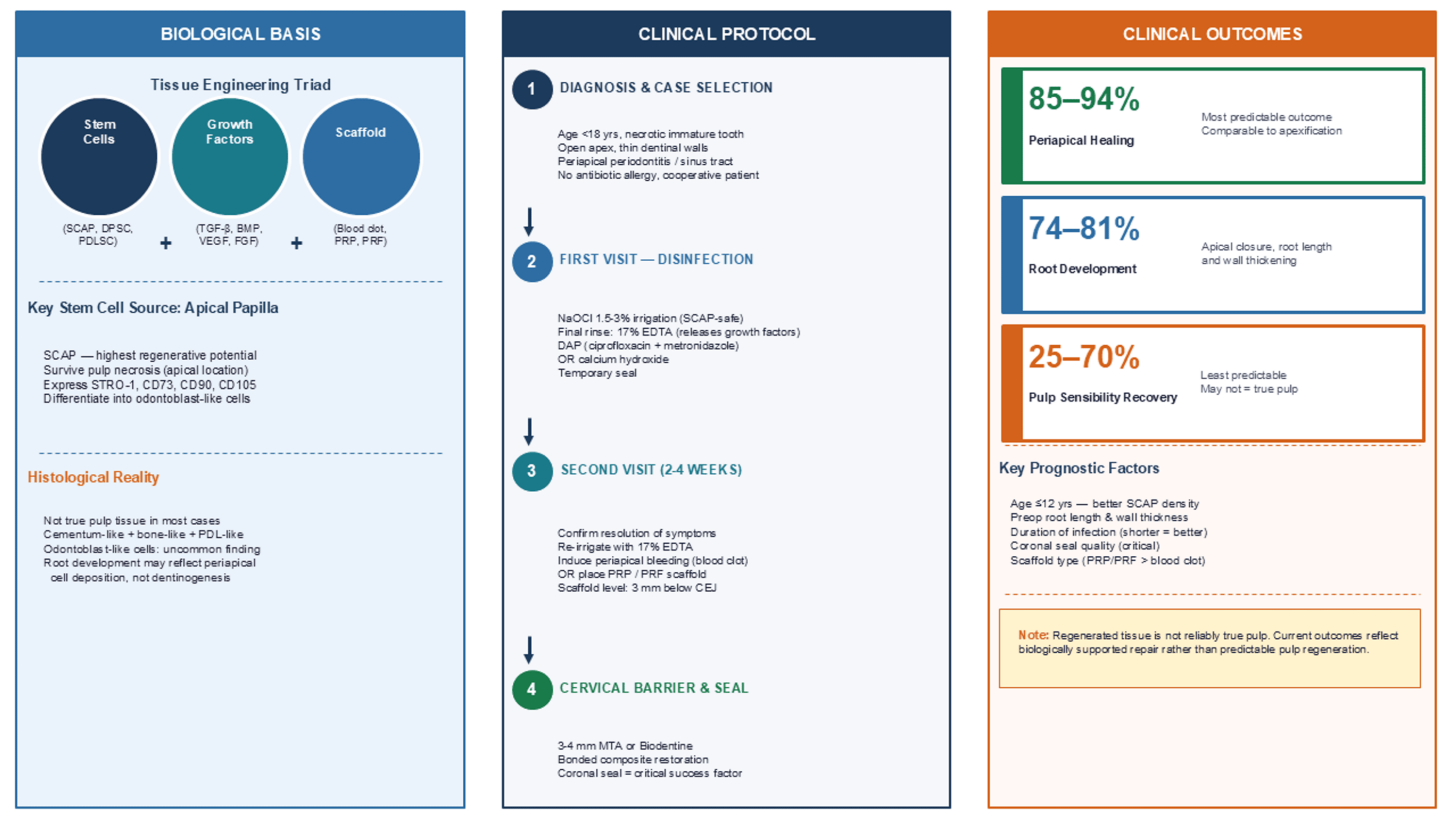
Biological Basis and Clinical Protocol of Regenerative Endodontic Procedures (REPs) Figure created by the author using PowerPoint (Microsoft Corporation, Redmond, USA). No generative AI features were used. SCAP: stem cells of the apical papilla; DPSC: dental pulp stem cells; PDLSC: periodontal ligament stem cells; PDL: periodontal ligament; TGF-β: transforming growth factor beta; BMP: bone morphogenetic protein; VEGF: vascular endothelial growth factor; FGF: fibroblast growth factor; PRP: platelet-rich plasma; PRF: platelet-rich fibrin; NaOCl: sodium hypochlorite; EDTA: ethylenediaminetetraacetic acid; DAP: double antibiotic paste; MTA: mineral trioxide aggregate; CEJ: cementoenamel junction.

Figure 1 summarizes the biological basis, clinical protocol, and reported outcomes of regenerative endodontic procedures.

Clinical outcomes and evidence synthesis

Representative Studies

Table 1 summarizes selected studies from the REP literature, chosen to reflect how the evidence has developed from early case reports to more recent controlled trials and meta-analyses. Studies were selected to represent key milestones, diverse study designs, and a range of patient populations and protocols, from the landmark 2004 case report by Banchs and Trope through to the most recent prospective cohort data and meta-analyses published up to 2025.

**Table 1:** Representative Clinical Studies on Regenerative Endodontic Procedures (2004-2024) TAP: triple antibiotic paste; DAP: double antibiotic paste; PRP: platelet-rich plasma; PRF: platelet-rich fibrin; mo: months; RCT: randomized controlled trial; SMD: standardized mean difference; REP: regenerative endodontic procedure; AAE: American Association of Endodontists; ESE: European Society of Endodontology; mo: months

Author (Year)	Study Design	Sample Size	Age (yrs)	Protocol	Follow-up	Key Outcome
Banchs & Trope (2004) [2]	Case report	2 teeth	11-12	TAP + blood clot	24 mo	Apical closure and root lengthening in both cases
Bose et al. (2009) [17]	Retrospective cohort	16 teeth	7-14	TAP + blood clot	12-48 mo	Root lengthening 81%; pulp sensibility recovery 56%
Jeeruphan et al. (2012) [18]	Retrospective cohort	45 teeth	6-18	REP vs apexification	24 mo	REP superior for root development; comparable periapical healing
Galler et al. (2016) [15]	Position statement	N/A	N/A	ESE guidelines	N/A	ESE protocol recommendations for revitalization procedures
Kahler et al. (2014) [16]	Prospective cohort	16 teeth	Mean 10	Blood clot scaffold	≥12 mo	Periapical healing 100%; root development in majority of cases
Alagl et al. (2017) [11]	Randomized controlled trial	40 teeth	9-16	PRP vs blood clot	36 mo	PRP: greater root length and wall thickness increases
Song et al. (2025) [19]	Retrospective cohort	85 teeth	6-18	AAE protocol	36 mo	Periapical healing 91%; root development 76%; calcification 22%
Swaikat et al. (2023) [20]	Systematic review & meta-analysis	32 studies	N/A	REP vs apexification	Variable	REP superior for root length and wall thickening
Theekakul et al. (2024) [21]	Prospective cohort	68 teeth	8-16	AAE protocol; blood clot	24-48 mo	Root development 79%; periapical healing 94%; calcification 18%

Synthesis of Outcomes

Periapical healing is the most consistent finding across REP studies, with reported rates ranging from 85% to 94% across individual studies in the literature [18,19,21]. This level of success likely reflects how well the disinfection phase controls the infection, rather than anything specific to the regenerative component, and it compares well with the healing rates reported after apexification. Root development outcomes, covering apical closure, root lengthening, and dentinal wall thickening, are seen in 74-81% of cases across the published literature, though with considerable variation between studies [17,22]. These figures represent ranges derived from individual studies rather than pooled estimates, and should be interpreted accordingly. A systematic review and meta-analysis by Swaikat et al. [20], which synthesized data from 32 studies comparing REPs with apexification, found that REPs produced greater root length and dentinal wall thickening, while both approaches achieved comparable periapical healing rates. Studies using PRP or PRF generally report better wall thickening results than those relying on a blood clot [11], which supports the view that a higher growth factor concentration in the scaffold benefits dentinal deposition.

Pulp sensibility recovery is the least predictable outcome, with published rates ranging from 25% to 70% [16]. The wide variation likely reflects true biological differences between cases rather than errors in how studies were conducted. A positive sensibility test after REP should be treated with caution since it may result from innervation of periapical tissues rather than from true intracanal nerve regeneration [23]. Clinicians should expect some variability in outcomes, especially when treating teeth with a history of prolonged infection or when patient cooperation is limited.

Prognostic Factors

Younger age at the time of treatment is one of the most consistently reported favorable prognostic factors. Patients aged 12 years and under tend to show better root development, probably because their apical papilla contains more viable SCAPs and the root still has more growth potential remaining [5,22]. Preoperative root dimensions also matter, since teeth with longer roots and thicker walls at baseline produce larger absolute gains [17]. A long history of infection before treatment is associated with poorer outcomes, likely because bacterial toxins reduce the viable SCAP population [14]. Poor coronal seal is one of the leading causes of treatment failure, as microleakage allows recontamination [16]. The choice of scaffold also influences outcomes, with PRP and PRF-based procedures generally showing better wall thickening than blood clot-based ones [10,11].

Limitations and challenges

Biological and Histological Limitations

The tissue that forms inside treated canals is not true pulp. Histological examination of retrieved REP-treated teeth has consistently shown a mixture of cementum-like, bone-like, and periodontal ligament-like tissue rather than an organized pulp-dentin complex [24]. Odontoblast-like cells lining the canal walls have been seen in some specimens, but this appears to be uncommon rather than the expected finding [8]. This matters for how we interpret radiographic data: continued root development or apical closure on a radiograph may be driven by cementum or bone deposition from periapical cells rather than by genuine dentinogenesis [25]. Clinicians should therefore not assume that radiographic improvement equals pulp regeneration, and should be careful not to overclaim what the procedure achieves biologically.

This histological reality has important long-term implications. If the intracanal tissue is predominantly cementum-like or periodontal in nature rather than functional pulp, the tooth may lack the dentinogenic protection and sensory warning system that native pulp provides. Whether this affects the long-term fracture resistance and survival of REP-treated teeth compared to teeth with healthy pulps or those treated with conventional apexification is a question that can only be answered with long-term clinical data, which are currently lacking.

Methodological and Clinical Challenges

The published literature on REPs is difficult to compare across studies because outcome definitions differ. Terms such as apical closure, root development, and success are not used consistently, and many studies report only short follow-up periods of 12-24 months [20,21]. Long-term data beyond five years are scarce. Most published work is retrospective, and patient-reported outcomes such as pain, satisfaction, and quality of life are rarely recorded. Crown discoloration from minocycline in TAP was a well-recognized problem in earlier protocols [7], though the shift to DAP and Biodentine has reduced this considerably [15]. Intracanal calcification develops in roughly 18-22% of treated teeth over the medium term [19] and can make retreatment difficult if the primary procedure fails. The use of antibiotic pastes in young patients also warrants ongoing consideration in the context of antimicrobial stewardship, and non-antibiotic alternatives such as photodynamic therapy are under investigation [7].

From a practical standpoint, REPs require patient cooperation across two appointments, careful handling of fragile thin-walled roots during instrumentation, and the ability to achieve a high-quality coronal seal. In younger pediatric patients, anxiety and limited cooperation can present real obstacles. The preparation of PRP or PRF also requires centrifugation equipment and trained personnel that may not be available in all clinical settings, limiting the adoption of enhanced scaffold protocols in resource-limited environments.

Clinical implications

Regenerative endodontic procedures should be the first treatment considered for necrotic immature permanent teeth in patients who meet the selection criteria. Before proceeding, the clinician needs to assess whether infection can be adequately controlled, whether the patient will attend both appointments reliably, and whether a good coronal seal can be achieved and maintained. When any of these conditions cannot be met, MTA apexification remains a well-supported alternative that should not be dismissed.

Patients and parents should be given honest information about what the procedure can and cannot achieve. This includes explaining that the tissue forming inside the canal may not be functional pulp, that sensitivity testing may remain negative even after successful treatment, that some degree of calcification can develop over time, and that long-term follow-up is needed. Managing expectations before treatment is more straightforward than dealing with disappointment afterward.

Future directions

The most active area of translational research involves delivering defined stem cell populations, above all SCAPs, directly into disinfected canals rather than relying on passive recruitment through bleeding [6]. Animal studies have shown that SCAP delivery on appropriate scaffolds can produce tissue with odontoblastic cells lining the canal walls, which more closely resembles true pulp than what is typically seen after standard clinical REP [26]. The main barriers to clinical use are the regulatory requirements for cell-based therapies and the practical challenges of harvesting and processing autologous cells. Dental stem cell banking from exfoliated deciduous teeth represents a potential future strategy for providing autologous stem cells for later use in regenerative procedures.

Engineered scaffolds that release growth factors at controlled rates are also under investigation. Injectable hydrogels, peptide-based matrices, and biodegradable polymers loaded with TGF-beta-1, VEGF, or BMP-2 have shown encouraging results in laboratory models by directing stem cells toward a pulpal rather than periodontal phenotype [8]. Chitosan-based nanofibrous scaffolds, for example, have demonstrated favorable biocompatibility, mechanical properties, and cell-supportive behavior in oral tissue engineering models, highlighting the potential of natural polymer-based matrices for regenerative dental applications [27]. These approaches address the main limitation of blood-clot and platelet-concentrate scaffolds, namely compositional variability between patients, by delivering defined concentrations of bioactive molecules in a reproducible manner. Self-assembling peptide nanofiber scaffolds have shown particular promise in preclinical models for supporting organized pulp-like tissue formation.

The standardization of outcome assessment is another important priority. Cone-beam computed tomography (CBCT)-based measurement of root dimensions provides more accurate and reproducible data than conventional periapical radiography, and its wider adoption in REP research would substantially improve comparability across studies [25]. Agreed-upon core outcome sets for REP trials, covering periapical healing, root development, pulp sensibility, patient-reported outcomes, and histological tissue characterization where possible, would enable more meaningful pooled analyses and meta-analyses than are currently achievable. Multicenter prospective studies with minimum five-year follow-up periods remain the most pressing evidence gap in the field.

## Conclusions

Regenerative endodontic procedures have changed how necrotic immature permanent teeth are managed. By addressing infection and creating conditions for continued root development, they offer outcomes that go beyond what apexification can achieve, with advantages for root wall thickening and length gain. The evidence supports their use as the treatment of first choice in appropriately selected cases, with consistently high rates of periapical healing and root development reported across diverse study populations.

At the same time, the limitations of current procedures deserve honest acknowledgment. The tissue that forms inside treated canals is not reliably true pulp, sensibility recovery is unpredictable, and long-term follow-up data beyond two years remain limited. Intracanal calcification and the challenges it creates for retreatment should be incorporated into the informed consent process.

What current REPs achieve may be better described as biologically supported repair rather than true pulp regeneration. Closing that gap will require advances in stem cell delivery, scaffold design, and growth factor control. Until those developments reach clinical practice, careful case selection, meticulous technique, a durable coronal seal, and long-term monitoring remain the foundations of successful treatment.

## References

[1] Andreasen JO, Farik B, Munksgaard EC (2002). Long-term calcium hydroxide as a root canal dressing may increase risk of root fracture. Dent Traumatol.

[2] Banchs F, Trope M (2004). Revascularization of immature permanent teeth with apical periodontitis: new treatment protocol?. J Endod.

[3] (2026). AAE clinical considerations for a regenerative procedure, revised 5/18/2021. https://www.aae.org/specialty/wp-content/uploads/sites/2/2021/08/ClinicalConsiderationsApprovedByREC062921.pdf.

[4] Murray PE, Garcia-Godoy F, Hargreaves KM (2007). Regenerative endodontics: a review of current status and a call for action. J Endod.

[5] Huang GT, Sonoyama W, Liu Y, Liu H, Wang S, Shi S (2008). The hidden treasure in apical papilla: the potential role in pulp/dentin regeneration and bioroot engineering. J Endod.

[6] Sonoyama W, Liu Y, Fang D (2006). Mesenchymal stem cell-mediated functional tooth regeneration in swine. PLoS One.

[7] Diogenes A, Henry MA, Teixeira FB, Hargreaves KM (2013). An update on clinical regenerative endodontics. Endod Top.

[8] Galler KM, D'Souza RN, Hartgerink JD, Schmalz G (2011). Scaffolds for dental pulp tissue engineering. Adv Dent Res.

[9] Torabinejad M, Turman M (2011). Revitalization of tooth with necrotic pulp and open apex by using platelet-rich plasma: a case report. J Endod.

[10] Shivashankar VY, Johns DA, Maroli RK, Sekar M, Chandrasekaran R, Karthikeyan S, Renganathan SK (2017). Comparison of the effect of PRP, PRF and induced bleeding in the revascularization of teeth with necrotic pulp and open apex: a triple blind randomized clinical trial. J Clin Diagn Res.

[11] Alagl A, Bedi S, Hassan K, AlHumaid J (2017). Use of platelet-rich plasma for regeneration in non-vital immature permanent teeth: Clinical and cone-beam computed tomography evaluation. J Int Med Res.

[12] Idris MI, Burhan AS, Hajeer MY, Sultan K, Nawaya FR (2024). Efficacy of the injectable platelet-rich fibrin (i-PRF) in gingival phenotype modification: a systematic review and meta-analysis of randomized controlled trials. BMC Oral Health.

[13] Trevino EG, Patwardhan AN, Henry MA, Perry G, Dybdal-Hargreaves N, Hargreaves KM, Diogenes A (2011). Effect of irrigants on the survival of human stem cells of the apical papilla in a platelet-rich plasma scaffold in human root tips. J Endod.

[14] Ruparel NB, Teixeira FB, Ferraz CC, Diogenes A (2012). Direct effect of intracanal medicaments on survival of stem cells of the apical papilla. J Endod.

[15] Galler KM, Krastl G, Simon S, Van Gorp G, Meschi N, Vahedi B, Lambrechts P (2016). European Society of Endodontology position statement: revitalization procedures. Int Endod J.

[16] Kahler B, Mistry S, Moule A, Ringsmuth AK, Case P, Thomson A, Holcombe T (2014). Revascularization outcomes: a prospective analysis of 16 consecutive cases. J Endod.

[17] Bose R, Nummikoski P, Hargreaves K (2009). A retrospective evaluation of radiographic outcomes in immature teeth with necrotic root canal systems treated with regenerative endodontic procedures. J Endod.

[18] Jeeruphan T, Jantarat J, Yanpiset K, Suwannapan L, Khewsawai P, Hargreaves KM (2012). Mahidol study 1: comparison of radiographic and survival outcomes of immature teeth treated with either regenerative endodontic or apexification methods: a retrospective study. J Endod.

[19] Song M, Jung HI, Kim SG (2025). Clinical outcomes of regenerative endodontic procedure: periapical healing, root development, and intracanal calcification. J Endod.

[20] Swaikat M, Faus-Matoses I, Zubizarreta-Macho Á (2023). Is revascularization the treatment of choice for traumatized necrotic immature teeth? A systematic review and meta-analysis. J Clin Med.

[21] Theekakul C, Banomyong D, Osiri S, Sutam N, Ongchavalit L, Jantarat J (2024). Mahidol study 2: treatment outcomes and prognostic factors of regenerative endodontic procedures in immature permanent teeth. J Endod.

[22] Alobaid AS, Cortes LM, Lo J (2014). Radiographic and clinical outcomes of the treatment of immature permanent teeth by revascularization or apexification: a pilot retrospective cohort study. J Endod.

[23] Diogenes A, Ruparel NB, Shiloah Y, Hargreaves KM (2016). Regenerative endodontics: a way forward. J Am Dent Assoc.

[24] Shimizu E, Jong G, Partridge N, Rosenberg PA, Lin LM (2012). Histologic observation of a human immature permanent tooth with irreversible pulpitis after revascularization/regeneration procedure. J Endod.

[25] Brochado Martins JF, Georgiou AC, Nunes PD, de Vries R, Afreixo VM, da Palma PJ, Shemesh H (2025). CBCT-assessed outcomes and prognostic factors of primary endodontic treatment and retreatment: a systematic review and meta-analysis. J Endod.

[26] Hargreaves KM, Giesler T, Henry M, Wang Y (2008). Regeneration potential of the young permanent tooth: what does the future hold?. J Endod.

[27] Al-Madhagy G, Alghoraibi I, Darwich K, Hajeer MY (2022). Evaluation of the chemical, morphological, physical, mechanical, and biological properties of chitosan/polyvinyl alcohol nanofibrous scaffolds for potential use in oral tissue engineering. Cureus.

